# Early-stage recovery of lithium from spent batteries via CO_2_-assisted leaching optimized by response surface methodology

**DOI:** 10.1038/s41598-024-67761-9

**Published:** 2024-07-29

**Authors:** Ksenija Milicevic Neumann, Muhammad Ans, Bernd Friedrich

**Affiliations:** IME – Process Metallurgy and Metal Recycling, RWTH Aachen University, 52056 Aachen, Germany

**Keywords:** Early-stage Li recovery, Spent NMC batteries, Carbonated H_2_O leaching, Response surface methodology, Optimized recycling efficiency., Chemical engineering, Materials for energy and catalysis

## Abstract

Recycling lithium (Li) from spent lithium-ion batteries (LIBs) due to the depletion of natural resources and potential toxicity is becoming a progressively favourable measure to realize green sustainability. Presently, the prevalent recycling technique relying on pyrometallurgy lacks the capability to extract lithium. Meanwhile, conventional hydrometallurgical processes frequently employ robust acidic solutions like sulfuric acid and precipitation agents such as sodium carbonate. Unfortunately, this approach tends to result in the extraction of lithium at the end of a lengthy process chain, leading to associated losses and creating challenges in managing complex waste. This study addresses a cost-effective and environmentally friendly early-stage lithium recovery from the thermally conditioned black mass. In this sense, a thermally conditioned black mass is subjected to the carbonization process in a water solution to transform the water-insoluble Li phase into soluble lithium bicarbonate (LiHCO_3_) and carbonate (Li_2_CO_3_) facilitating its selective separation from other elements. Response surface methodology (RSM)—a statistical tool integrated with central composite design (CCD) is employed to optimize the parameters for Li recovery. Temperature, solid–liquid (S/L) ratio, leaching time and CO_2_ flow rate are considered as variable factors in modelling the optimum recycling process. A quadratic regression model is developed for Li recovery and based on ANOVA analysis, (S/L) ratio, temperature and time are identified as statistically significant factors. Experimental results demonstrate a maximum leaching efficiency of lithium with optimized parameter set, achieving a recovery rate of 97.18% with a fit response of 93.54%.

## Introduction

Presently, the demand for lithium-ion batteries (LIBs) as electrochemical power sources, driven by their widespread use in electric vehicles, mobile and smartphones, and other applications due to long-life cycles, high energy density, and low self-discharge is higher than ever before. Considering the global market growth of LIB products, it is expected that a large number of spent LIBs will be increased^1–5^. For instance, cylindrical (NMC-18650) and prismatic cells are the most popular types for electronic and automotive applications^6^. However, spent LIBs pose severely human health and environmental risks due to the presence of various organic chemicals and heavy metals^7–10^. Despite, spent LIBs contain valuable metals, such as nickel (Ni), cobalt (Co), and lithium (Li), underscoring the high economic value^11,12^. Therefore, recycling and treatment of spent LIBs have become dominant and imperative from the viewpoint of ecological protection and resource preservation.

The recycling of Li garners significant attention due to the substantial environmental impact associated with primary production from natural resources^13^ and potential supply risk^14^. To date, the exploration of metallurgical Li recovery methods from spent LIBs have covered both pyrometallurgical and hydrometallurgical techniques and their combination^15–22^. However, the pyrometallurgical routes exhibits notable drawbacks, such as the emission of toxic gasses that contributes to air pollution, high energy consumption, and significant Li losses as it becomes part of the slag system and flue dust^23^.

The conventional pyrometallurgical recycling process for lithium-ion batteries entails subjecting them to high-temperature smelting, resulting in the recovery of nickel, cobalt, and copper in the form of an alloy. However, crucial battery materials such as lithium, aluminum, and iron become constituents of a generated slag, rendering their further extraction economically unviable. Notably, the combustion of graphite from the anode during this process contributes to carbon dioxide emissions. Subsequent to the smelting phase, the alloy derived necessitates additional hydrometallurgical processing involving multiple steps to recuperate salts suitable for reuse in battery production. Therefore, hydrometallurgy emerges as a viable alternative, gaining extensive traction in both industrial applications and academic research due to its high metal recovery efficiency and cost-effectiveness. Before hydrometallurgical treatment, lithium-ion batteries are mechanically shredded, electrolyte evaporated, plastic and metallic housing material separated by diverse screening methods. The most critical elements (lithium, nickel, cobalt and graphite) coming from cathode and anode are obtained in form of black powder called black mass. Traditional hydrometallurgical methods for processing of black mass and recovery of these critical materials often rely on strong acids, posing severe environmental and human health issues. The most common processing route of black mass to recover lithium goes through multiple stages starting from dissolution of all metals with sulphuric acid and removal of graphite. The obtained solution is further undergoing the processing via precipitation, cementation and other methods to remove the impurities—aluminium, iron and copper. Such a purified sulphuric solution containing nickel, cobalt, manganese and lithium is entering the solvent extraction method, in which after multiple stage extraction, nickel, cobalt and manganese are recovered. Lithium is recovered at the end from the leftover solution by precipitation adding sodium carbonate to form lithium carbonate and waste stream of sodium sulphate. Owing to the extensive processing pathway, lithium is undergoing substantial losses, resulting in the generation of multiple waste streams^24,25^

As an alternative, methods like the carbonation process have been explored to convert insoluble solid Li-compounds into H_2_O-soluble compounds, mitigating these environmental and economic concerns^26–28^. This process was established based on the kinetic reactions of spent electrodes with CO_2_ solution by means of ion exchange extraction under different conditions. The dissolution rate of lithium carbonate (Li_2_CO_3_) exponentially increased with increasing CO_2_ flow rate, with key reactions during the process are expressed in Eqs. (1–4), followed by the precipitation of Li_2_CO_3_ through solution heating^28,29^.

1 $$CO_{2} + \, H_{2} O \leftrightarrows H_{2} CO_{3}  \leftrightarrows HCO_{3}^{-} + \, H^{ + } \leftrightarrows \, CO_{3}^{2 - } + \, 2H^{ + }$$

2 $$Li_{2} CO_{3} + \, H^{ + } \to 2Li^{ + } + \, HCO_{3}$$

3 $$Li^{ + } + \, HCO_{3} \leftrightarrows Li^{ + } + \, HCO_{3}^{-} \leftrightarrows LiHCO_{3}$$

4 $${\text{Li}}_{{2}} {\text{CO}}_{{3}} + {\text{ H}}_{{2}} {\text{CO}}_{{3}} \to {\text{2LiHCO}}_{{3}}$$

Schwich et al.^18^ investigated an effective eco-friendly “Early-Stage Lithium Recovery” (ESLR) method involving Li leaching through carbonation with supercritical CO_2_ in a cost-intensive autoclave process, achieving an efficiency of 79%. In contrast, developing a cost-efficient carbonation process under atmospheric pressure possess a high potential in tackling the drawbacks of this approach. So far, no systematic research has explored the optimal parameters, such as, leaching time, temperature and S/L ratio in CO_2_-assisted hydrometallurgy under atmospheric pressure, particularly using the statistical design of experiments for selectively Li recovery.

Response surface methodology (RSM) is a statistical technique used for collecting functional relationship between influential factors and adequate response. It establishes predict response values of multivariant experimental design to determine process optimization. The RSM evaluates an appropriate operating condition which is significantly reported in the literature^30,31^. Furthermore, due to diagnostic or screening studies to locate ideal settings in the experimental design, this modelling approach is suitable for implementing the quadratic polynomial model^32^. Therefore, RSM's analytical and experimental processes are typically more advanced and modern than any other modelling technique^33,34^.

Considering process-related challenges posed by conventional recovery methods, specifically extraction of Li, this study proposed an early-stage Li recovery process from spent LIBs using environmentally friendly green hydrometallurgy i.e., CO_2_-H_2_O leaching under atmospheric pressure. It comprises low-cost and eco-friendly carbonation processes at the early stages of process chain, diverging from traditional acidic leaching or smelting^15,24,25^. Using RSM, a statistical modelling technique is employed to assess the efficiency of lithium leaching and validate operational parameters, aiming to minimize losses typically encountered during the multi-step precipitation chain for separating Cu, Fe/Al, Mn and Ni/Co. Quadratic regression models are employed for each variable involved in the leaching process, enabling the exploration of interaction effects among several factors and the determination of significance/insignificance of various terms. Laboratory trials are conducted to verify these findings. The proposed Li recovery process outlined in this study is based on a fundamental structure, incorporating well-defined particle size distribution and scientifically optimized solid/liquid ratios. It serves as a benchmark in the field and has the potential to catalyze advancements in industry by reducing toxic waste and increasing Li recovery rates.

## Experimental

### Material preparation & leaching method

Spent NMC cells have been pyrolyzed at 600 ℃ under vacuum. Subsequently, the material underwent shredding in a cutting mill and sorted to obtain black mass sieved to particles < 1 mm in size. These initial steps were conducted by an external company. The obtained black mass was further milled to reduce the particle grain size less than 63 µm, enhancing the interaction of liquid solution with solid during leaching and thus improving the Li recovery yield. A planetary ball mill (*PKM—Pulverisette 6, FRITSCH GmbH, Germany*) with a stainless steel vail (400 mL in volume) was used for the milling process. The stainless steel vail was filled with 2/3rd of powder and 14 balls of 20 mm diameter were introduced for the milling process. The rotational speed was set at 450 rpm and the black mass milled for 5 min, as reported in the previous literature^35^. Subsequently, dry sieve analysis was conducted using a sieving tower (*AS200, RETSCH GmbH, Germany*) to collect particles sized < 63 μm. During the sieve analysis, a frequency of 2.0 mm/g with an interval of 30 s was maintained for 2 min.

**Figure 1:**
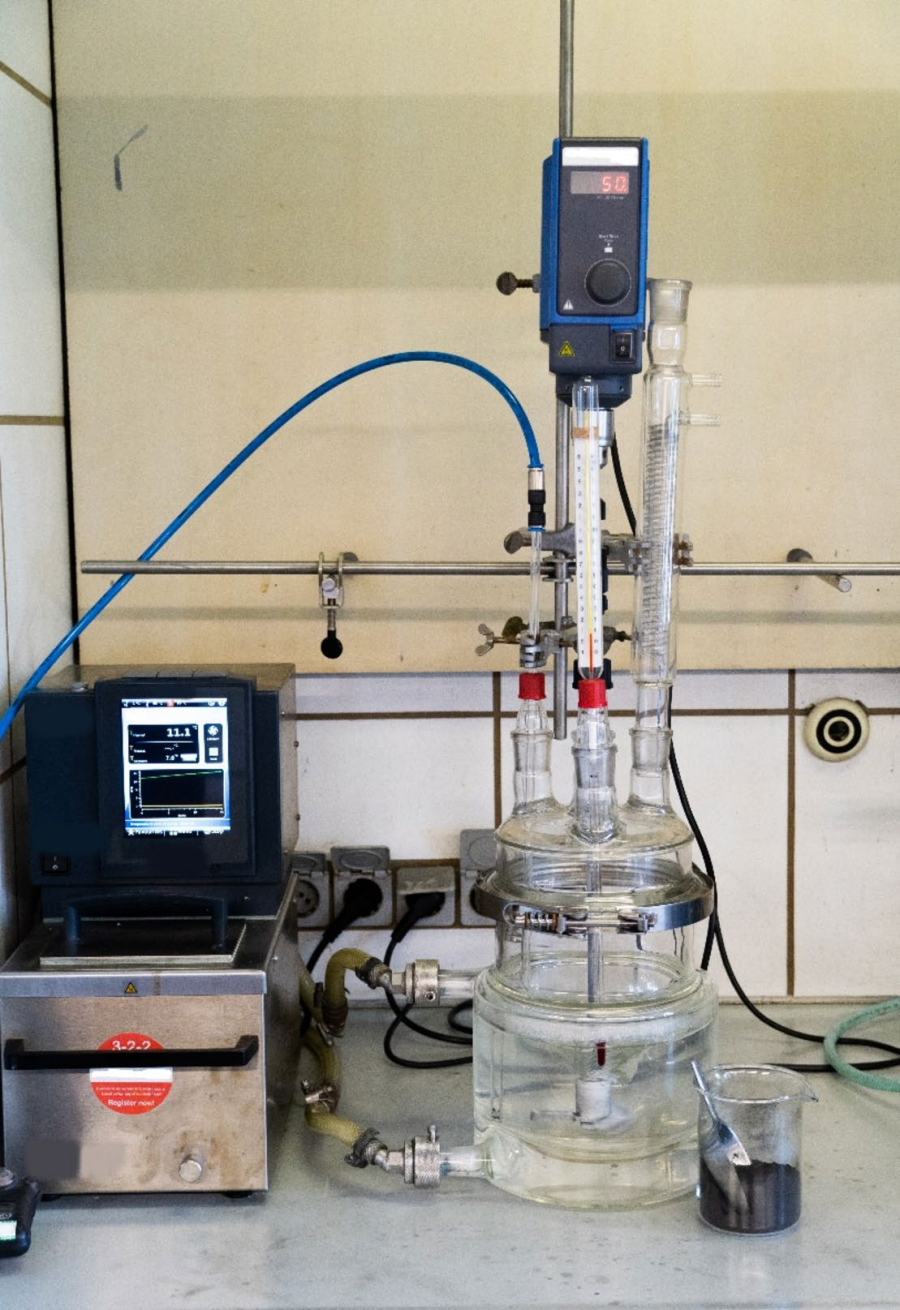
Hydrometallurgical setup for carbonation process and lithium recovery from the black mass.

All leaching processes were conducted in deionized (DI) water with a continuous flow of CO_2_. The leaching setup consisted of a four-neck round-bottomed reactor of double-wall, connected to a heating bath circulation thermostat (*Huber CC-304B, Kältemaschinenbau AG, Germany*). The desired amount of black mass dissolved into a 1.5 L water solution and stirred at a constant rate of 350 rpm with a mechanical stirrer throughout all experiments. A CO_2_ glass lance (Ø10 mm outer diameter) was immersed into the solution through the lid and a thermometer was used to measure the reactor's temperature. The entire setup is depicted in Fig. 1. Following leaching, solid residue filtration was performed on a suction funnel using *Macherey–Nagel MN-619 ¼* filter paper. The solid residue was collected and dried in a heating furnace at 80 ℃ for 12 h. Finally, the filtered Li solution was boiled to precipitate the Li-carbonate in a solid state. Before precipitation, samples of the leached solution were taken for chemical analyses to determine Li concentration, i.e., recovery rate.

The concentration of Li in the solution sample has been determined by ion-selective electrode (*Mettler Toledo, DX207-Li)* and further confirmed through the inductively coupled plasma optical emission spectroscopy (ICP-OES) method (*Ciros Vision, Spectro Analytical Instruments GmbH, Germany*). The lithium leaching efficiency (η_Li_) has been calculated using the Eq. (5):

5 $$\eta Li \left[ \% \right] = \frac{{cLi\left[ \frac{mg}{L} \right]* V\left[ L \right]}}{{mBM \left[ {mg} \right]* \nu Li}}*100$$

where c_Li_ is the measured concentration of Li with ion selective electrode in obtained solution after leaching, V is volume of the reaction mixture, m_BM_ is the mass of the input material (black mass), ν_Li_ is the percentage of Li in input material (black mass) measured by ICP-OES method.

### Optimization of leaching parameters by response surface methodology (RSM) and central composite design (CCD)

The aim of optimization is to maximize Li yield from the spent black mass while minimizing the experimental trials. To achieve this, we employed a statistical modelling tool (known as RSM) with a central composite design (CCD). This approach allows us to evaluate the uniform precision design within various parameters and reduce prediction errors. CCD is widely used to optimize variables based on multivariant nonlinear regression models derived from the appropriate experimental parameters. It enables the assessment of adequate operating conditions and facilitates the interactions of various parameters influencing the process^32^. The CCD technique acquires experimental values for fitting the model (a second-order model also known as the rotatable variance model). In our study, the set of four variable parameters are temperature (10–77 ℃), time (10–180 min), solid–liquid (S-L) ratio (10:1—70:1 g/L), and CO_2_ flow (3–6 L/min) were considered in this analysis. The stirring rate and particle size remained constant, as these parameters were deemed to have a lower impact compared to others and were not varied significantly by other researchers^18^ in the field, based on prior experience. The parameter ranges were selected based on consideration such as solubility of CO_2_ and lithium carbonate, equipment capabilities and existing literature. Additionally, the Li-yield (η_Li_ [%]) was chosen as the response variable. A two-level factorial design was employed to determine appropriate parametric conditions corresponding to maximum predicted values. The two-level factorial in the statistical modelling was achieved as 31 (= 2^ k^ + 2 k + 7), where k is the number of factors = 4, to ensure randomness and avoid biases. Table 1 shows the parametric levels with coded and uncoded values. To verify the reproducibility and reliability of the optimum parameters, an additional experiment was conducted using defined parameters to demonstrate Li yield via leaching. The coefficient correlation (R^2^) values indicated the polynomial fit. The RMS and CCD technique were implemented using *MINITAB 19.0* software, facilitating graphical analysis, desirability functions and optimizer plots.

**Table 1:** Uncoded parameter levels used in the leaching of Li.

Variable parameters		
Parameters with units	Low values	High values
Temperature (℃)	10	77
Leaching time (min)	10	180
S-L ratio (g L^-1^)	10:1	70:1
CO_2_ flow rate (L min^-1^)	3.0	6.0

Constant parameters		
Agitation rate (rpm)	350	
Particle size (μm)	< 63	
Volume of reaction mixture (L)	1.5	

### Material characterization

**Figure 2:**
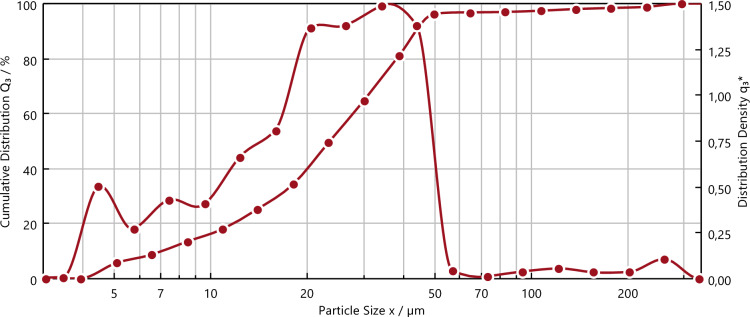
Density and distribution of particles size of the input material.

**Figure 3:**
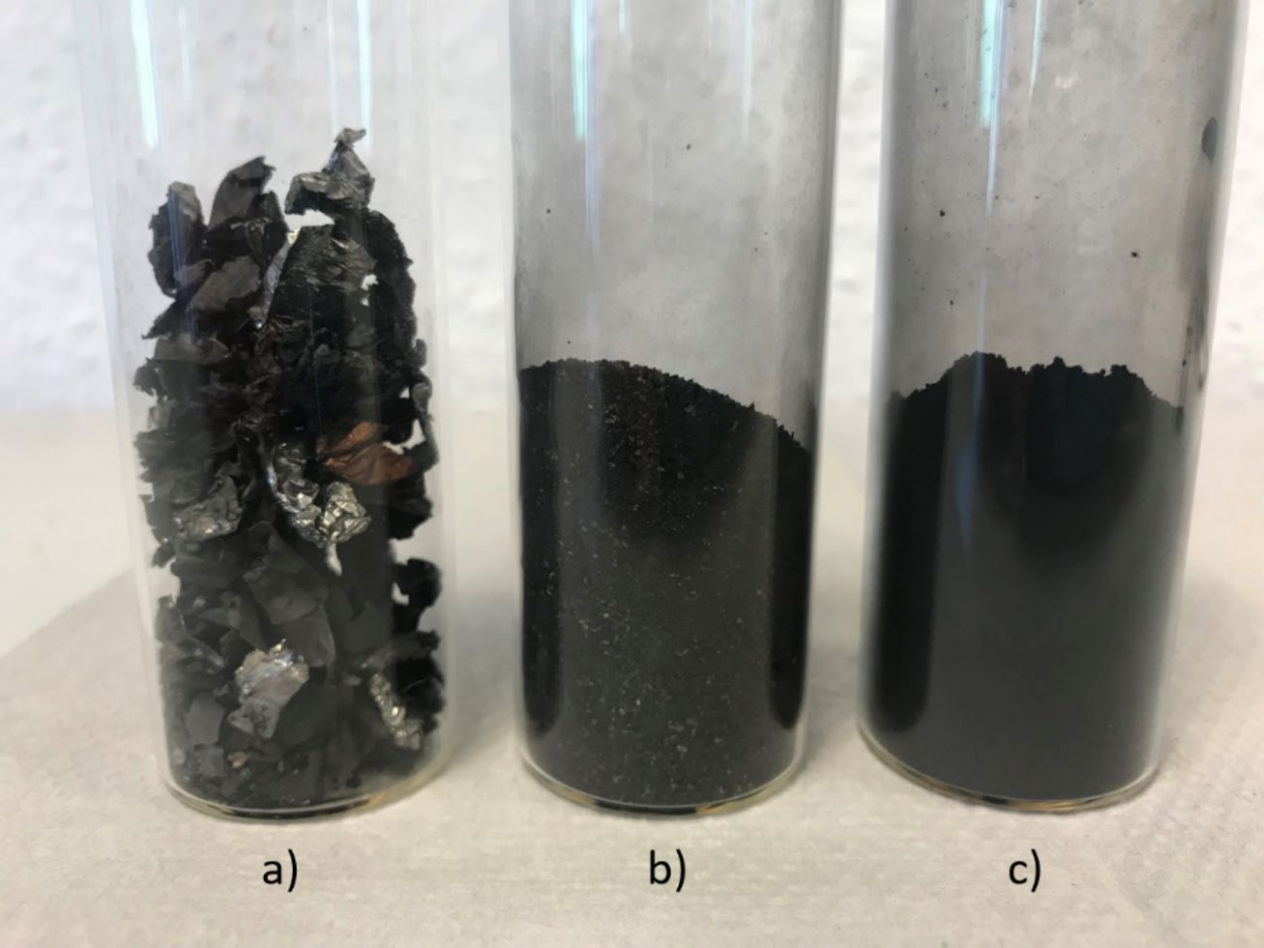
Black mass fractions: (**a**) > 500 µm, (**b**) 500 µm < x > 125 µm (**c**) < 63 µm.

The black mass used in this investigation originates from thermally and mechanically pre-treated NMC cells, which is further milled to particle size below 63 µm. Dynamic image analysis has been performed to determine the average particle size of the prepared input material (*QuickPick Oasis, Sympatec GmbH, Clausthal-Zellerfeld, Germany*). As shown in Fig. 2, over 90.3% of particles were found to be smaller than 45.27 µm, with 50.3% measuring below 23.55 µm. This indicates that after ball milling and sieving processes, the desired particle fractions < 63 µm were successfully obtained. Also, the decision to use lower particle fraction was influenced by the noticeable presence of current collectors (Cu and Al foils) in coarser size, as shown in Fig. 3. These foils pose a hinderance to Li extraction, underscoring the importance of selecting finer particle sizes for improved processing efficiency.

The ICP-OES method determined the chemical composition and the percentage of elements in the input mixture (see Table 3). The fluorine contents are analysed by combustion-ion chromatography (CIC)—A1 combustion-IC, while the carbon contents are assessed by total carbon analysis (TC) via Analytic Jena multi N/C 2001 S instrument.

The ICP-OES analysis of the input material was performed twice on the residual Li samples, and the medium value of 2.67 wt.-% of Li was taken for yield calculations (Eq. 5). X-ray diffraction (XRD) was carried out to evaluate the crystalline phases of the powder samples. The crystalline phases (i.e., C, LiF, Ni , Mn_0.95_O, CoO) were identified when powder diffraction treated by *HighScore Plus, Malvern Panalytical B.V*

## Results and discussion

### Model development by central composite design (CCD) for the leaching process

A total of 31 experimental trials of Li leaching (Table 2) were carried out using various regression models via CCD (MINITAB® software). Table 3 illustrates the analysis of Li (Y_Li_)—carried out with ICP-OES. The design of experiments (DOE) is essential for completing statistical analysis and verifying the correctness of variables (coded/uncoded). Therefore, each response was fitted by the second-order multivariable polynomial (as provided by the regression, Eq. 6) to showcase the accuracy and reliability of the results.

6 $${\text{Response}} = {88}.{57} - 0.{44}0 {\text{Temperature}} - 0.0{342} {\text{Time}} - 0.{7}0{4} {\text{S}} - {\text{L}} {\text{ratio }} - {2}.{29} {\text{CO}}_{{2}} {\text{flow }} + 0.00{317} {\text{Temperature}} \times {\text{Temperature}} + 0.00{365} {\text{S}} - {\text{L}} {\text{ratio}} \times {\text{S}} - {\text{L}} {\text{ratio}} + 0.00{1692} {\text{Temperature}} \times {\text{Time }} + 0.0{329} {\text{Temperature}} \times {\text{CO}}_{{2}} {\text{flow }} + 0.00{655} {\text{Time}} \times {\text{CO}}_{{2}} {\text{flow}} + 0.0{156} {\text{S}} - {\text{L}} {\text{ratio}} \times {\text{CO}}_{{2}} {\text{flow}}$$

**Table 2:** Experiments with different combinations of four factors using CCD along with experiments and predicted values of all responses (time, leaching time, S-L ratio and CO2 flow rate) where the experiment with highest response is highlighted in bold.

StdOrder	RunOrder	Temp (^o^C)	Leaching time (min)	S-L ratio (g/L)	CO_2_ flow rate (L min^-1^)	Experimental response (Y_Li_) (%)	Predicted response (Y_P_)	Percent relative error (RE,%)	Mean absol. Error (MAE)	Root mean sq. error (RMSE)
27	1	10	180	70:1	6.0	48.37	49.82	3.00	1.45	2.10
23	2	77	10	10:1	3.0	70.26	66.14	5.86	4.12	16.97
18	3	77	95	40:1	4.5	65.81	63.47	3.55	2.34	5.46
6	4	10	10	70:1	6.0	50.40	45.72	9.30	4.69	21.95
4	5	45	95	40:1	4.5	47.76	56.21	17.68	8.44	11.29
13	6	45	95	40:1	7.5	61.90	57.81	6.61	4.09	16.73
20	7	45	95	40:1	1.5	57.74	54.60	5.43	3.14	9.83
14	8	45	10	40:1	4.5	61.07	50.59	17.16	10.48	19.84
25	9	45	95	20:1	4.5	67.07	64.23	4.23	2.84	8.06
12	10	45	95	40:1	6.0	59.40	57.01	4.03	2.40	5.74
16	11	10	95	40:1	4.5	61.61	54.72	11.19	6.89	47.50
15	12	77	180	70:1	6.0	75.81	70.91	6.46	4.89	23.95
5	13	45	265	40:1	4.5	72.89	67.45	7.47	5.44	29.65
17	14	45	95	100:1	4.5	53.29	48.99	8.06	4.29	18.44
11	15	10	10	70:1	3.0	45.34	48.10	6.09	2.76	7.61
8	16	77	180	70:1	3.0	66.57	62.10	6.72	4.47	20.00
24	17	45	95	40:1	4.5	59.40	56.21	5.38	3.20	10.22
7	18	77	10	70:1	3.0	50.49	44.50	11.85	5.98	35.79
19	19	45	95	40:1	4.5	58.39	56.21	3.74	2.18	4.76
1	20	10	180	10:1	3.0	76.67	69.80	8.95	6.86	47.12
21	21	45	95	40:1	4.5	59.38	56.21	5.34	3.17	10.05
22	22	10	180	10:1	6.0	69.29	67.91	1.99	1.38	1.91
2	23	10	10	10:1	6.0	67.42	63.81	5.36	3.61	13.03
**30**	**24**	**77**	**180**	**10:1**	**6.0**	97.18	89.00	8.42	8.18	16.88
28	25	10	180	70:1	3.0	51.19	48.16	5.91	3.02	9.14
29	26	10	10	10:1	3.0	71.91	69.74	3.02	2.17	4.72
26	27	77	10	70:1	6.0	48.19	49.28	2.26	1.09	1.19
31	28	77	10	10:1	6.0	68.86	67.37	2.17	1.49	2.23
9	29	77	180	10:1	3.0	85.99	83.74	2.62	2.26	5.09
3	30	45	95	40:1	3.0	57.93	55.41	4.35	2.52	6.35
10	31	45	95	10:1	4.5	68.36	69.30	1.37	0.94	0.88
								Mean	**2.97**	**3.74**

**Table 3:** ICP-OES, CIC and TC analysis of one random sample of input material < 63 µm—black mass in wt.-%.

C	Ni	Co	Mn	Fe	Al	Li	Cu	F	P
46.7	6.00	5.54	6.46	2.76	3.00	2.65	3.46	2.53	0.83

To further evaluate the development of the model for several indicator performances Eqs. (7–10) are employed^30,31^

7 $${\text{Coefficient of determination}:R}^{2}=1-\frac{\sum_{i=1}^{N}{\left({Y}_{Li}-{Y}_{P}\right)}^{2}}{\sum_{i=1}^{N}{\left({Y}_{Li}-{\overline{Y} }_{Li}\right)}^{2}}$$

8 $$\text{Percent Relative error}:RE(\text{\%})=\left|\frac{{Y}_{Li}-{Y}_{P}}{{Y}_{Li}}\right|\times 100$$

9 $$\text{Mean absolute error}:MAE=\frac{{\sum }_{i=1}^{N}\left|{Y}_{Li}-{Y}_{P}\right|}{N}$$

10 $$\text{Root mean square error}:RMSE=\sqrt{\frac{\sum_{i=1}^{N}{\left({Y}_{Li}-{Y}_{P}\right)}^{2}}{N}}$$

where, *Y*_*Li*_ is the experimental response, *N* is the total number of trails. The low values of root mean sq. root (RMSE) (< 3.8) and mean absolute error (MAE) (< 3.0) indicate that the model’s predictions closely align with the experimental values.

**Figure 4:**
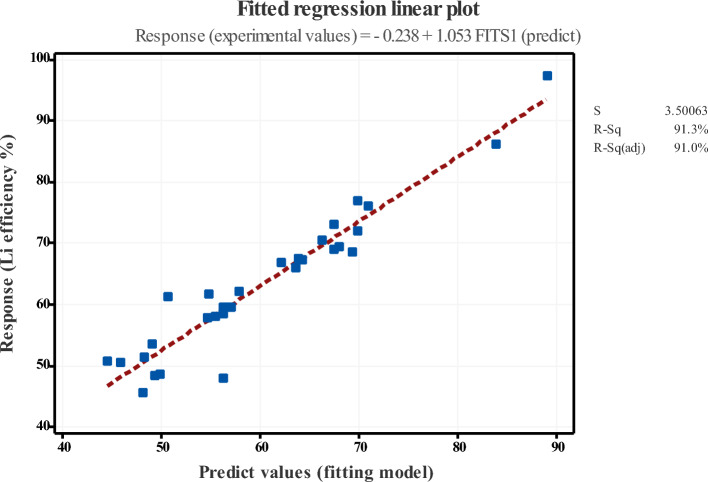
Fits regression plot of experimental and predicted response (S = standard deviation, R-sq (adj) = adjusted R^2^, R-sq (pred) = predicted R^2^).

The orthogonal design yields approximation model values, resulting in a less complex and reliable analysis. Figure 4 shows the regression plot of experimental data (vs. predicted response). The R^2^ value of the experimental response (91.30%) exceeds that of the predicted value (75.47%) underscoring the importance of optimization. ANOVA analysis (at a 5% significance level) confirms the quadratic model's importance and identifies the effect of each parameter. The p- and F-values assess the significance of each coefficient in the parameter set for the correlations between the numerical values (see Table 4). These values assess the decision of the statistical model, providing crucial evidence against the null hypothesis. Notably, the p-values < 0.05 (except for CO_2_ flow) indicate satisfactory model performance, as deviations from the null hypothesis are negligible in standard distribution data^36^.

**Table 4:** ANOVA analysis of variance of Li recovery by leaching.

Source	DF	Adj SS	Adj MS	F-Value	P-Value
Model	10	3732,52	373,25	21,24	0,000
Linear	4	3231,88	807,97	45,99	0,000
Temperature	1	463,66	463,66	26,39	0,000
Time	1	770,09	770,09	43,83	0,000
S-L ratio	1	1973,17	1973,17	112,31	0,000
CO2 flow	1	9,72	9,72	0,55	0,466
Square	2	354,69	177,35	10,09	0,001
Temperature*Temperature	1	84,74	84,74	4,82	0,040
S-L ratio*S-L ratio	1	158,61	158,61	9,03	0,007
2-Way Interaction	4	434,53	108,63	6,18	0,002
Temperature*Time	1	371,69	371,69	21,16	0,000
Temperature*CO2 flow	1	43,76	43,76	2,49	0,130
Time*CO2 flow	1	11,14	11,14	0,63	0,435
S-L ratio*CO2 flow	1	7,93	7,93	0,45	0,509
Error	20	351,39	17,57		
Lack-of-Fit	16	251,97	15,75	0,63	0,772
Pure Error	4	99,42	24,86		
Total	30	4083,92			

**Figure 5:**
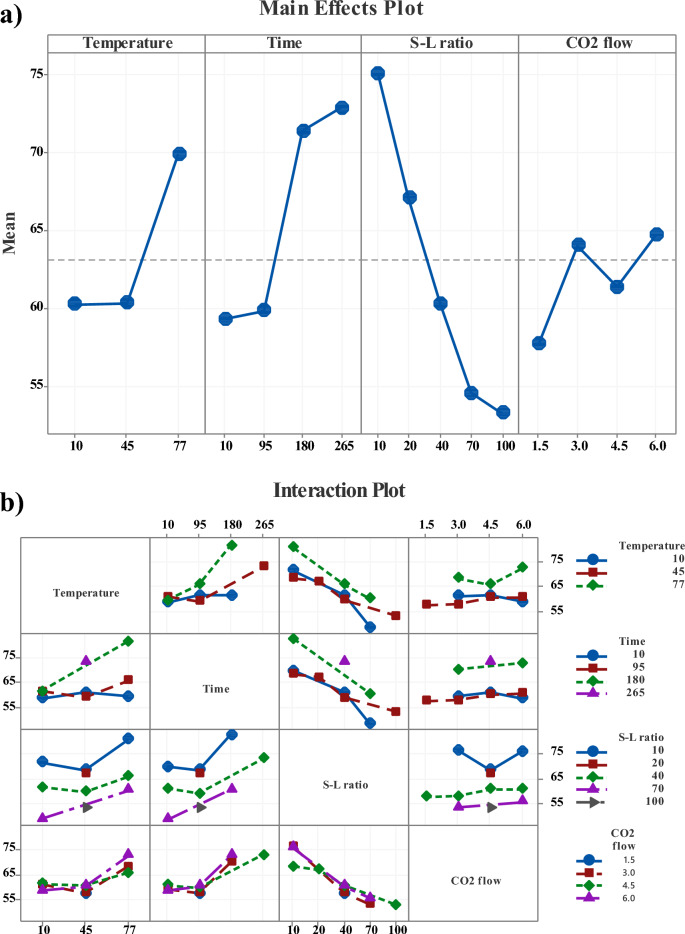
(**a**) Main effect and (**b**) interaction plots for responses of optimum parameters.

Main effect plots containing experimentally fitted data are used to assess the yield of Li during the leaching process—see Fig. 5. In Fig. 5a, it is observed that leaching efficiency remains relatively unchanged at lower temperature ranges (10 to 45 ℃). However, a significance increase in Li efficiency is noted at higher temperatures (77 ℃). In the case of time, a slight increase of Li efficiency is observed (up to 95 min), followed by a plateau reached at 180 min, which persists until 265 min. Although CO_2_ flow has a minimal impact on the leaching rate, a slight increase is observed at 6 L min^-1^ with time dependency. Notably, varying S-L ratios drastically enhance the leaching behaviour, with higher efficiency observed at a ratio of 10:1 g/L, which decreases as the ratio increases. Figure 5b.shows the interaction plots depicting individual leaching efficiency as a function of responses. All interactions involving CO_2_ flow are parallel with the x-axis confirming this parameter's lack of interaction effect with Li recovery efficiency. Other plots show varying degrees of interaction between the effects, with some showing nonlinear trends. The interaction effect of leaching temperature vs. time shows excessive tendency values at 77 °C and 180 min, respectively. A significantly higher response is observed for a lower S-L ratio in the interaction plot of S-L ratio vs. temperature and time. Based on the interaction graphs, maximum leaching rates are determined at a low S-L ratio, coupled with high temperature and time.

**Figure 6:**
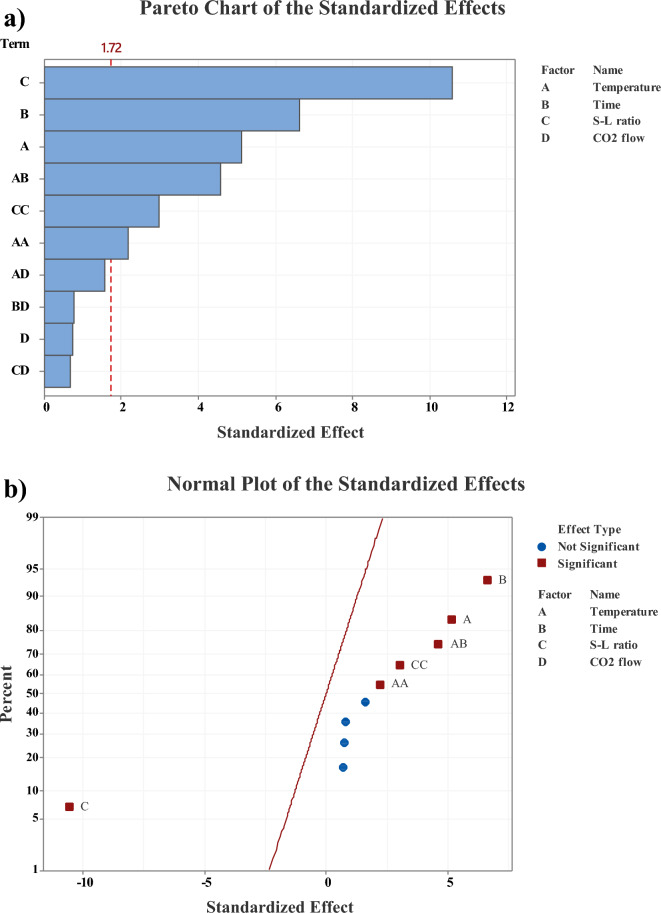
(**a**) The Pareto and (**b**) Normal plot of the standardized effect of variables (temperature, time, S-L ratio, and CO_2_ flow), as a function of efficiency response, α = 0.1.

Pareto and normal diagrams were designed for leaching efficiencies with all responses to support the validity and satisfactory approximation of the created model (see Fig. 6.) A reference line (red line in Fig. 6a) passes through the main effects with the highest magnitude starting with S-L ratio (C), then time (B) and temperature (C). However, this reference line does not intersect with the CO_2_ flow (D) main effect, indicating that this variable may not significantly influence the leaching processes.

The residuals of all variables are positioned near the diagonal line (Fig. 6b), suggesting the normal data distribution function. The red squares represent the significant effects contributing to maximum efficiency, whereas the blue points express a non-significant effect. As can be seen, the S-L ratio exhibits a significant impact, positioned furthest from the 0 on the x-axis, confirming its prominence in the Pareto chart (Fig. 6 a). Its location on the negative side of the x-axis suggests that the response decreases with a shift from low to high values of this factor. On the other hand, time and temperature demonstrate a positive effect, indicating an increase in the response as a factor value transitions from low to high. Also, significant interactions between temperature and time (AB) and squared terms for S-L ratio (CC) and temperature (AA) are evident.

The reduction of the nominal terms in the regression Eq. (6) and the confidence level increase was tested to improve the model’s reliability. Despite these adjustments, no significant changes were revealed, confirming the high conformity of the applied model and obtained results.

### Contour plots of lithium leaching

**Figure 7:**
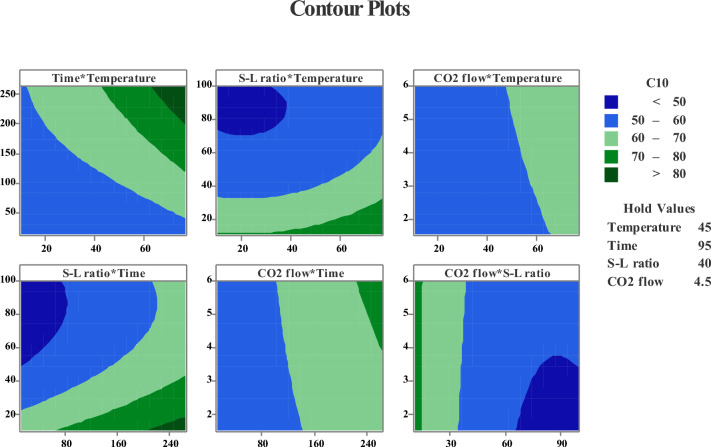
Contour plots as a function of response.

2D contour plots provide a comprehensive depiction of the relationship between central composite values' responses and the variables of the resulting models, as shown in Fig. 7. The curved contours signify the inclusion of statistically significant quadratic terms in the model, as observed in previous sections. The contour plots are obtained only for central values (hold values in Fig. 7) and represent how the efficiency rate changes with variations in parameters. The bell shape (the core of the counterplots), enable the direct establishment of maximum efficiency between the time*temperature, S/L ratio*temperature, and S/L ratio*time can be predicted. According to Fig. 7, temperature and S-L ratio are supreme factors in Li extraction. The Li recovery rates experience significant enhancement (from 50 to 80%) with prolonged time (> 180 min) and elevated temperature (> 60 ℃) at a minimum S-L (10:1). However, the influence of CO_2_ flow*S-L ratio, CO_2_flow*time and CO_2_flow*temperature remain limited. These findings align with previous explanations, indicating that CO_2_ flow and interactions of CO_2_ flow with other variables do not exert a substantial influence the Li recovery rates.

### Desirability function of lithium recovery

The contour plots could not determine the desired optimum conditions for the exact parameters. To address this, the desirability function plots were generated to fine-tune the appropriate model with optimal conditions. This approach effectively validated the model using various experimental variables to achieve the key trade-offs. Based on the multiple response and desirability function, the optimum parameters of maximum Li efficiency are defined as 77 ℃, 180 min, S-L ratio (10:1) g/L, and CO_2_ flow rate of 6.0 L/min. The fitted result confirms that these values yield an approximate response of ~ 93.54%, as detailed in Table 5.

**Table 5:** Desirability function outcome.

Solution	Temperature (^o^C)	Time (min)	S-L ratio (g/L)	CO_2_ flow (L/min)	Response Fit (%)	Composite desirability
1	77	180	10:1	6	93.5394	0.929799

**Figure 8:**
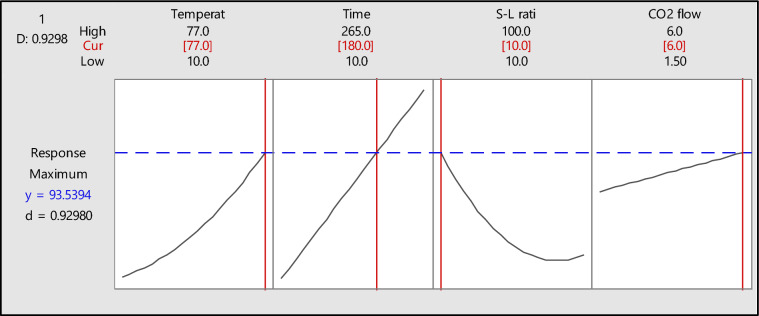
Response optimization diagrams for leaching parameters.

The program combines individual desirability data into a single composite number, further maximizing the function. The composite desirability for Li leaching was defined as 0.92980, aiming for an efficiency of ~ 97% (Table 5), indicating the attainment of maximum yield. In Fig. 8, the blue dotted lines represent the maximum efficiency obtained based on the optimum conditions (highlighted in red), which were used in the experimental trials.

The acceptable desirability reported by Resentera et al.^37^ was ~ 0.95 for optimizing Li extraction at low temperatures. In our study, the achieved composite desirability values are ~ 0.93, validating our analytical approach and confirming the actual response. The critical phase reaction requires sufficient dispersion of S-L mass transfer to dissolve solid particles in a CO_2_-assisted water solution. Consequently, adequate time is necessary for achieving uniform dispersion and S-L equilibrium of the particles during leaching. This observation is further supported by the favourable effect of immersion duration on Li dissolution in H_2_O.

### Effect of operating conditions as a function response

The optimum efficiency of Li recovery from NMC powder was determined at a S-L ratio of 10:1 (g L^-1^) by introducing carbonated water. As depicted in Fig. 5, the behaviour of Li leaching interactions and main effect plots with optimized variables predicted by RSM and verified by lab-based experimental trials. The results show that the Li_2_CO_3_ conversion improved with decreasing solid concentration from 70:1 (~ 75% leaching efficiency) to 10:1 (~ 97% leaching efficiency), taking the same temperature, retention time and gas flow parameters. The carbonation process, involving the conversion of Li^+^ into the CO_2_-water, follows a typical noncatalytic three-phase reaction (gas–liquid-solid)^28^. Increasing solid concentration in the mixture leads to higher bulk diffusion resistance at the S-L boundary and liquid phase, hindering complete dissolution in the reaction^38^. Besides, higher solid concentration reduces the interfacial reaction area of the particles due to increased powder density and friction between solid phases^39^. Consequently, decreased mixing efficiency results in difficulty in gas–liquid mass transfer difficulty, slowing down the reaction and resulting to lower Li^+^ conversion. This suggests that an excess of H_2_O is required for high Li^+^ dissolution in the system.

The investigation into the effect of temperature and time on Li recycling revealed a notable enhancement in efficiency with varying temperature ranges, as shown in Fig. 5. Specifically, the result indicated that the Li recovery rate increased from 69 to 97% by increasing the temperature from 10 ℃ to 77 ℃ for 180 min. This observation was made with a S/L ratio of 10:1 and a CO_2_ flow of 6 l/min. Clearly, the higher temperature ranges are more favourable for Li^+^ dissolution. The literature shows diverse findings on this subject, Yi et. al.^24^ reported better efficiencies at lower temperatures, Zhang et. al.^27^ observed a slight increase in efficiency with rising temperatures, while Makuza et. al.^40^ noted an increase in efficiency rates with higher leaching temperatures. Although the solubility of Li_2_CO_3_ and CO_2_ generally decreases with temperature^41,41^. Makuza’s^40^ hypothesis suggests that higher temperatures lead to enhancement of reaction kinetics. Also, higher CO_2_ concentration at lower temperatures enforces reaction by increasing the pH values of the solution. However, its important to note that a lower pH value of the solution can result in the simultaneous dissolution of other heavy metals, consequently leading to lower Li recovery efficiency. Indeed, our experiments analyzed higher concentrations of other metals (such as Mn, Ni, Co etc.,) in leached solution at lower temperatures, thus confirming these findings.

Furthermore, achieving optimal S-L mass transfer and phase dispersion during the carbonation reaction necessitates sufficient time. Specifically, the system must reach an equilibrium where particles are uniformly distributed, and S-L equilibrium is established before carbonation. In our research, it was observed that efficient carbonation was achieved at 180 min, indicating a positive impact on the dissolution of Li_2_CO_3_.

The effect of the CO_2_ flow rate is shown in Fig. 5a. It is evident that increasing the CO_2_ flow does not exhibit a clear trend or significant impact on the leaching rate. However, according to the optimisation method, the higher CO_2_ flow rates resulted in increased Li concentration in the water solution. This phenomenon aligns with the findings of Yi et. al.^43^ and is contributed to the augmentation of the mass transfer volumetric coefficient in the gas–liquid phases. Despite the statistical evaluation not showing a straightforward tendency, the higher CO_2_ flow rate is beneficial in increasing the stirring effect and, herewith better interaction between gas–liquid-solid phases.

### Confirmation of reproducibility of experiments

**Figure 9:**
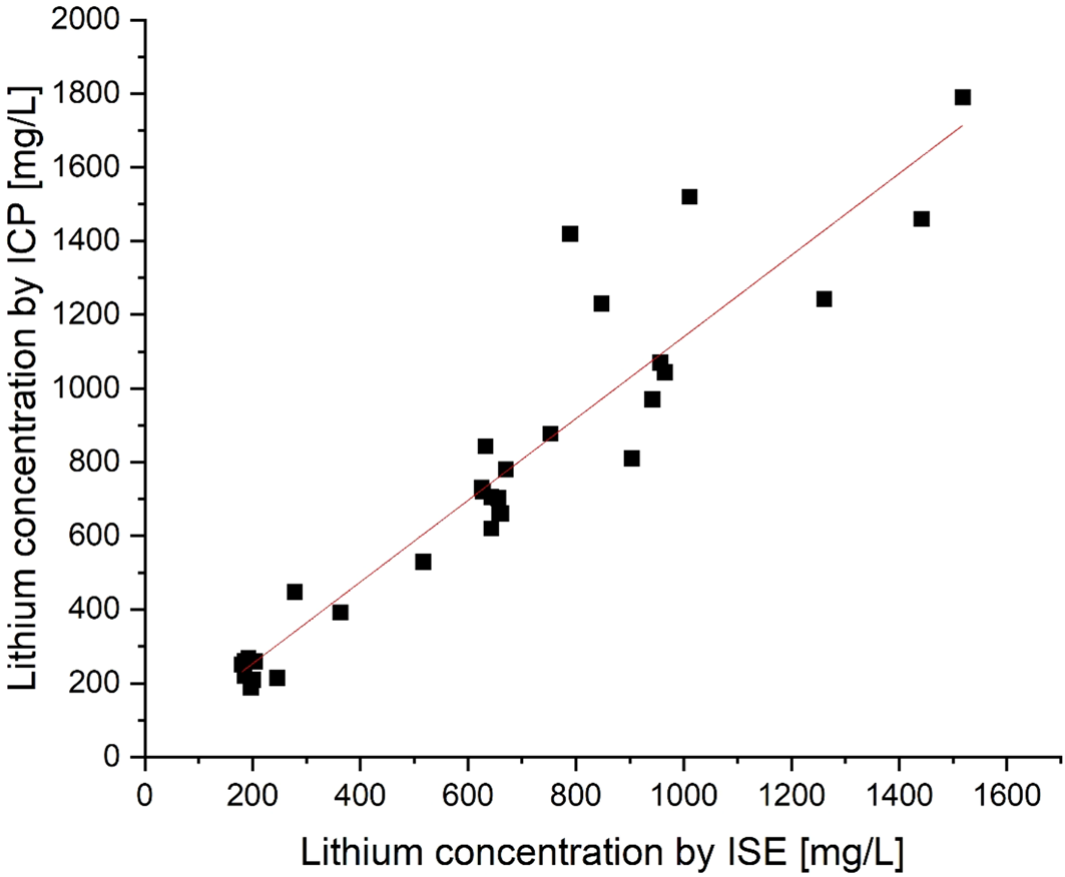
Lithium concentration measured by ISE and ICP-OES methods.

To validate the obtained experimental results, the trial with the highest Li recovery efficiency (77 ℃, 180 min. 10:1 g/L, 6 l/min) was repeated 5 times, yielding an average efficiency value of 94.73%. This value closely aligns with both our experimental and statistically optimized efficiency rates. Also, reference tests were conducted using the same optimal parameters (77 ℃, 180 min. 10:1 g/L) but without CO_2_ injection, repeated multiple times, with an average value of 59.08%. This further supports the effectiveness of the carbonation process, which improves the Li leaching efficiency by 35.65%. Furthermore, the Li concentration in solution after the carbonation process was analyzed by two analytical methods, (1) ion selective electrode (ISE) and (2) ICP-OES. The values obtained from both analyses are plotted against each other in Fig. 9. The Pearson coefficient R is 0.93875, indicating a strong linear correlation between the values obtained from both analyses.

## Conclusion

This study provides a novel approach to achieve high-efficiency recovery of spent LIBs using environmentally friendly and acid-free hydrometallurgical methods. We investigated the effect of temperature, time, S-L ratio, and CO_2_ flow on effective lithium recovery from spent batteries, using modelling techniques such as RSM and CCD. Experimental findings aligned closely with the predicted values obtained by quadratic and statistical models. Temperature, time and S-L ratio emerged as the significant factors in ANOVA analysis, contributing prominently to achieving the highest Li leaching efficiency, whereas the impact of CO_2_ flow rate on Li recovery was comparatively less pronounced. The optimized conditions yielding the highest Li yield of 97.18% and a response fit of 93.54% were determined at 77 ℃, 180 min, S-L ratio (10:1) g/L, and CO_2_ 6.0 L min^-1^. The significant value of composite desirability (0.92980) of the predicted model suggests the reliability and precision of the optimum combination for all responses. Further investigation will explore the feasibility of translating these laboratory-scale results into the semi-pilot scale operations, including considerations for a reactor volume of 100 L.

## Data Availability

Data generated or analysed during this study are mainly included in this published article. Any additional data required will be provided upon request by corresponding author.

## References

[1] Steward, D., Mayyas, A. & Mann, M. Economics and challenges of li-ion battery recycling from end-of-life vehicles. *Proced. Manufact.* **33**, 272–279 (2019).

[2] Armand, M. *et al.* Lithium-ion batteries – Current state of the art and anticipated developments. *J. Power Sour.* **479**, 228708 (2020).

[3] Wang, X., Gaustad, G., Babbitt, C. W. & Richa, K. Economies of scale for future lithium-ion battery recycling infrastructure. *Resour. Conserv. Recycl.* **83**, 53–62 (2014).

[4] Richa, K., Babbitt, C. W., Gaustad, G. & Wang, X. A future perspective on lithium-ion battery waste flows from electric vehicles. *Resour. Conserv. Recycl.* **83**, 63–76 (2014).

[5] Xu, C. *et al.* Future material demand for automotive lithium-based batteries. *Commun. Mater.*10.1038/s43246-020-00095-x (2020).

[6] Gordon C. C. Yang, Yu-Chen Huang & Shengyi Huang. Recovery of valuable metals from cylindrical 18650-type spent lithium-ion batteries. *The 15th Japan/Korea Int. Symp. Resour. Recycl. Mater. Sci.* 1–10 (2017).

[7] Lebedeva, N. P. & Boon-Brett, L. Considerations on the chemical toxicity of contemporary li-ion battery electrolytes and their components. *J. Electrochem. Soc.* **163**, A821–A830 (2016).

[8] Kang, D. H. P., Chen, M. & Ogunseitan, O. A. Potential environmental and human health impacts of rechargeable lithium batteries in electronic waste. *Environ. Sci. Technol.* **47**, 5495–5503 (2013). 23638841 10.1021/es400614y PMC5920515

[9] Boyden, A., Soo, V. K. & Doolan, M. The environmental impacts of recycling portable lithium-ion batteries. *Proced. CIRP* **48**, 188–193 (2016).

[10] Mrozik, W., Rajaeifar, M. A., Heidrich, O. & Christensen, P. Environmental impacts, pollution sources and pathways of spent lithium-ion batteries. *Energ. Environ. Sci.* **14**, 6099–6121 (2021).

[11] Ma, X., Ma, Y., Zhou, J. & Xiong, S. The recycling of spent power battery economic benefits and policy suggestions. *IOP Conf. Ser. Earth Environ. Sci.* **159**, 12017 (2018).

[12] Lander, L. *et al.* Financial viability of electric vehicle lithium-ion battery recycling. *iScience* **24**, 102787 (2021). 34308293 10.1016/j.isci.2021.102787 PMC8283134

[13] Jiang, S. *et al.* Environmental impacts of lithium production showing the importance of primary data of upstream process in life-cycle assessment. *J. environ. Manag.* **262**, 110253 (2020). 10.1016/j.jenvman.2020.110253 32250776

[14] Meng, F., McNeice, J., Zadeh, S. S. & Ghahreman, A. Review of lithium production and recovery from minerals, brines, and lithium-ion batteries. *Miner. Process. Extr. Metall. Rev.* **42**, 123–141 (2021).

[15] Träger, T., Friedrich, B. & Weyhe, R. Recovery concept of value metals from automotive lithium-ion batteries. *Chemie Ingenieur Technik* **87**, 1550–1557 (2015).

[16] Georgi-Maschler, T., Friedrich, B., Weyhe, R., Heegn, H. & Rutz, M. Development of a recycling process for Li-ion batteries. *J. Power Sour.* **207**, 173–182 (2012).

[17] Peters, L., Schier, C. & Friedrich, B. *Mineralische Nebenprodukte und Abfälle,* (eds) by E. Thomé-Kozmiensky*, et al.* (Thomé-Kozmiensky Verlag GmbH, 2018), pp. 338–359.

[18] Schwich, L., Schubert, T. & Friedrich, B. Early-stage recovery of lithium from tailored thermal conditioned black mass part I: Mobilizing lithium via supercritical Co2-carbonation. *Metals* **11**, 177 (2021).

[19] Wang, H. & Friedrich, B. Development of a highly efficient hydrometallurgical recycling process for automotive li–ion batteries. *J. Sustain. Metall.* **1**, 168–178 (2015).

[20] Ferreira, D. A., Prados, L. M. Z., Majuste, D. & Mansur, M. B. Hydrometallurgical separation of aluminium, cobalt, copper and lithium from spent Li-ion batteries. *J. Power Sour.* **187**, 238–246 (2009).

[21] Lee, C. K. & Rhee, K.-I. Reductive leaching of cathodic active materials from lithium ion battery wastes. *Hydrometallurgy* **68**, 5–10 (2003).

[22] Meshram, P., Abhilash, B. D. P., Mankhand, T. R. & Deveci, H. Acid baking of spent lithium ion batteries for selective recovery of major metals: A two-step process. *J. Ind. Eng. Chem.* **43**, 117–126 (2016).

[23] Sommerfeld, M. *et al.* A combined pyro- and hydrometallurgical approach to recycle pyrolyzed lithium-ion battery black mass part 1: Production of lithium concentrates in an electric arc furnace. *Metals* **10**, 1069 (2020).

[24] Zhou, Z., Lai, Y., Peng, Q. & Li, J. Comparative life cycle assessment of merging recycling methods for spent lithium ion batteries. *Energies* **14**, 6263 (2021).

[25] Chen, M. *et al.* Recycling end-of-life electric vehicle lithium-ion batteries. *Joule* **3**, 2622–2646 (2019).

[26] Hu, J., Zhang, J., Li, H., Chen, Y. & Wang, C. A promising approach for the recovery of high value-added metals from spent lithium-ion batteries. *J. Power Sour.* **351**, 192–199 (2017).

[27] Zhang, J., Hu, J., Zhang, W., Chen, Y. & Wang, C. Efficient and economical recovery of lithium, cobalt, nickel, manganese from cathode scrap of spent lithium-ion batteries. *J. Clean. Prod.* **204**, 437–446 (2018).

[28] Yi, W., Yan, C., Ma, P., Li, F. & Wen, X. Refining of crude Li2CO3 via slurry phase dissolution using CO2. *Sep. Purif. Technol.* **56**, 241–248 (2007).

[29] Jandová, Jitka, Dvořák, Petr, Kondás, Ján. & Havlák, Lubomír. Recovery of lithium from waste materials. *Ceram. Silik.* **56**(1), 50–54 (2012).

[30] Myers, R. H., Montgomery, D. C. & Anderson -Cook, C. M. *Response surface methodology. Process and product optimization using designed experiments* (Wiley, 2016).

[31] Sarabia, L. A., Ortiz, M. C. & Sánchez, M. S. Response surface methodology. In *Comprehensive Chemometrics* (ed. Walczak, B.) 287–326 (Elsevier, 2020).

[32] Irfan, M. F. *et al.* Modeling and optimization of aqueous mineral carbonation for cement kiln dust using response surface methodology integrated with box-behnken and central composite design approaches. *Min. Metall. Explor.* **37**, 1367–1383 (2020).

[33] de Oliveira, L. G. *et al.* Response surface methodology for advanced manufacturing technology optimization: Theoretical fundamentals, practical guidelines, and survey literature review. *Int. J. Adv. Manuf. Technol.* **104**, 1785–1837 (2019).

[34] Bashir, M. J. K., Amr, S. S. A., Aziz, Shuokr Qarani, NgChoon, Aun & Sethupathi, Sumathi. Wastewater treatment processes optimization using response surface methodology (RSM) compared with conventional methods: Review and comparative study. *Middle East J. Sci. Res.* **23**, 244–252 (2015).

[35] Hadef, F. & Ans, M. X-ray analysis and Rietveld refinement of ball milled Fe50Al35Ni15 powder. *Surf. Interfaces* **26**, 101303 (2021).

[36] Carpinteyro-Urban, S. & Torres, L. G. Use of response surface methodology in the optimization of coagulation- flocculation of wastewaters employing biopolymers. *Int. J. Environ. Res.* **7**(3), 717–726 (2013).

[37] Resentera, A. C., Esquivel, M. R. & Rodriguez, M. H. Low-temperature lithium extraction from α-spodumene with NH4HF2: Modeling and optimization by least squares and artificial neural networks. *Chem. Engi. Res. Des.* **167**, 73–83 (2021).

[38] Plummer, L. N., Parkhurst, D. L. & Wigley, T. M. L. Critical review of the kinetics of calcite dissolution and precipitation. In *Chemical Modeling of Aqueous Systems* (eds Jenne, E. A. & Jenne, E. A.) 537–573 (American Chemical Soc, 1979).

[39] Zhang, S., Li, T., Zhu, B. & Zhu, Z. Gas-liquid mass transfer in three-phase mechanical agitated reactor. *J. Chem. Ind. Eng.* **56**, 220–226 (2015).

[40] Makuza, B., Yu, D., Huang, Z., Tian, Q. & Guo, X. Dry grinding—carbonated ultrasound-assisted water leaching of carbothermally reduced lithium-ion battery black mass towards enhanced selective extraction of lithium and recovery of high-value metals. *Res. Conserv. Recycl.* **174**, 105784 (2021).

[41] Miller, R. R., Smith, S. H. & Williams, D. D. Solubility of lithium carbonate at elevated temperatures. *J. Chem. Eng. Data* **16**, 74–75 (1971).

[42] Lucile, F. *et al.* Solubility of carbon dioxide in water and aqueous solution containing sodium hydroxide at temperatures from (293.15 to 393.15) K and Pressure up to 5 MPa: Experimental measurements. *J. Chem. Eng. Data* **57**(784), 789 (2012).

[43] Yi, W.-T., Yan, C.-Y. & Ma, P.-H. Kinetic study on carbonation of crude Li2CO3 with CO2-water solutions in a slurry bubble column reactor. *Korean J. Chem. Eng.* **28**, 703–709 (2011).

